# Climate change, rodent ecology, and the rising risk of hantavirus outbreaks: an alarming public health concern

**DOI:** 10.3389/fmicb.2026.1943856

**Published:** 2026-09-18

**Authors:** Saurabh RamBihariLal Shrivastava, Prateek Sudhakar Bobhate, Harshal Mendhe, Nandkishor Bankar, Ashwini Tidake, Prithvi Petkar

**Affiliations:** 1 Department of Community Medicine, Datta Meghe Medical College, Datta Meghe Institute of Higher Education and Research, Nagpur, Maharashtra, India; 2 Dr. D. Y. Patil School of Allied Health Sciences, Dr. D. Y. Patil Vidyapeeth, Pune (Deemed to be University), Pimpri, Pune, Maharashtra, India; 3 Department of Community Medicine, All India Institute of Medical Sciences, Vijaypur, Jammu, India; 4 Department of Microbiology, Datta Meghe Medical College, Datta Meghe Institute of Higher Education and Research, Nagpur, Maharashtra, India

**Keywords:** climate change, climate-sensitive infectious diseases, hantavirus, one health, rodent ecology, viral evolution, zoonotic spillover

## Abstract

The Anthropocene era has been characterized by a higher risk of zoonotic viral outbreaks, which often result from a combination of factors, such as climate change, biodiversity loss, land-use patterns, and increased opportunities for human–animal interactions. The current perspective provides an integrated synthesis of how climate change-driven alterations in rodent ecology impact the molecular evolution, transmission dynamics, and emergence of hantaviruses, with an emphasis on the underlying virological mechanisms and their implications for One Health preparedness. Hantaviruses have undergone extensive diversification throughout their evolution and host adaptation and can infect rodents, bats, reptiles, and fish. Rodents serve as the principal natural reservoirs for hantaviruses, with many viral species exhibiting long-term evolutionary associations with specific rodent host species. Climate change has been recognized as one of the key determinants for the emergence of hantavirus outbreaks in different parts of the world. Climate-related factors may facilitate viral amplification through multiple mechanisms, including their impact on evolution, mutation, and reassortment. To conclude, climate change has shaped the ecology, transmission dynamics, and molecular evolution of hantaviruses by altering rodent behavior, their habitats, and the environmental persistence of viral particles. Climate-induced ecological disruption has created opportunities for viral amplification within reservoir hosts and augmented the risk of zoonotic spillover into humans. These insights provide a conceptual framework for anticipating future outbreaks in the context of climate change and support the integration of ecological, virological, and public health surveillance systems. There is an urgent need to ensure that virological preparedness strategies evolve in accordance with changing environmental conditions by strengthening genomic surveillance and integrated One Health frameworks.

## 1 Introduction

In the current Anthropocene era, an unprecedented rise in the risk of zoonotic viral outbreaks and epidemics has been reported, often driven by climate change, biodiversity loss, human activities that alter the natural landscape, and increased opportunities for human–animal interactions (Plowright et al., 2021; Sánchez et al., 2021). A rise in the probability of the emergence of infectious diseases with epidemic or pandemic potential has been reported in the last few decades due to environmental disruption, which has increased spillover from wildlife reservoirs to human populations (Plowright et al., 2021). With increasing climate-sensitive viral outbreaks, there is a crucial need to understand the ecological determinants of virus transmission and the necessity to adopt integrated One Health surveillance frameworks (Stephens et al., 2021). In fact, artificial intelligence-based predictive models have estimated that climate-induced shifts in wildlife distribution can facilitate novel cross-species viral transmission in the future (Grange et al., 2021).

Among zoonotic pathogens, hantaviruses have attracted significant scientific attention because they are highly adaptable rodent-borne RNA viruses capable of causing severe diseases, namely, hemorrhagic fever with renal syndrome and hantavirus cardiopulmonary syndrome, in humans (Munir et al., 2021). Moreover, changing climatic conditions, such as varying rainfall patterns, warmer temperatures, and ecological instability, can influence fluctuations in reservoir populations of rodents and create more opportunities for hantavirus transmission to humans (Vial et al., 2023). Recent studies and developments have improved our understanding of the ecology of the causative viruses, environmental determinants, and surveillance approaches (Munir et al., 2021; Ramos-Nino et al., 2026; Vial et al., 2023). However, the majority of studies have focused on exploring climate variability, rodent population dynamics, viral evolution, and public health preparedness as separate domains (Fabbri et al., 2025; Grange et al., 2021; Munir et al., 2021; Ramos-Nino et al., 2026; Stephens et al., 2021; Vial et al., 2023). The current perspective provides an integrated synthesis of how climate change-driven alterations in rodent ecology impact the molecular evolution, transmission dynamics, and emergence of hantaviruses, highlighting the underlying virological mechanisms and their implications for One Health preparedness.

## 2 Hantaviruses: virological and molecular overview

Hantaviruses belong to the family Hantaviridae (order Bunyavirales), which includes multiple genera and several species, the majority of which are pathogenic to humans (Kuhn and Schmaljohn, 2023). These viruses have shown extensive diversification in their evolution and host adaptation as they can infect rodents, bats, reptiles, and fish (Bradfute et al., 2024). The virus has a tri-segmented negative-sense single-stranded RNA genome consisting of small, medium, and large segments (Riquelme, 2021). This segmented genome is crucial for facilitating genetic reassortment, thereby aiding the evolution of the virus and the emergence of novel variants (Kim et al., 2021). The small segment encodes the nucleocapsid protein, which is vital for encapsidating RNA, stabilizing the genome, and regulating antiviral signaling in the host (Kell, 2022). Similarly, the large segment encodes the RNA-dependent RNA polymerase responsible for the transcription and replication of the virus (Bradfute et al., 2024). Envelope glycoproteins form tetrameric spikes on the viral envelope and mediate receptor binding and membrane fusion during host-cell entry (Bradfute et al., 2024; Kim et al., 2021). Accordingly, any structural alterations in these glycoproteins can affect viral infectivity, tissue tropism, and host adaptation potential (Bradfute et al., 2024).

The nucleocapsid protein safeguards viral RNA and enables ribonucleoprotein complex formation, which is essential for replication (Munir et al., 2021). The nucleocapsid protein also modulates host innate immune pathways by interfering with mechanisms responsible for signaling interferons (Kell, 2022). Once hantaviruses enter mammalian host cells, they replicate in the cytoplasm, where viral RNA transcription and translation occur using host ribosomes and viral polymerase complexes (Munir et al., 2021). The newly synthesized virions assemble within the Golgi apparatus before they are released via exocytosis (Koehler et al., 2022). To gain entry into host cells, hantaviruses utilize β3 integrins and other endothelial receptors, and they have high tropism for vascular endothelial cells, resulting in capillary leakage, pulmonary edema, and injury to renal vessels (Koehler et al., 2022; Munir et al., 2021). These viruses evade the host immune system by suppressing innate antiviral signaling and interfering with interferon production. However, in the case of rodent reservoirs, persistent infection often results from immune modulation, which limits excessive inflammatory responses, thereby permitting the persistence of the virus (Kell, 2022).

## 3 Rodent reservoir ecology and viral persistence

Rodents have been identified as natural reservoirs for hantaviruses, with distinct viral species linked with rodents through long-term adaptation (Cabrera et al., 2023). In fact, this co-divergence between the causative virus and its reservoir host has contributed to the genetic stability and ecological persistence of the virus across diverse geographic ecosystems (Milholland et al., 2019). As reservoir rodents tend to remain asymptomatic despite chronic infection, there is sustained viral circulation without any detectable mortality in these hosts (Zuo et al., 2021). The virus can establish persistent infection in rodents by modulating innate and adaptive immune responses, thereby avoiding excessive inflammatory damage (Kell, 2022). This alters interferon signaling and enhances immunoregulatory cytokine activity, contributing to the persistence of the virus in in rodent hosts (LaPointe et al., 2023). At the same time, persistent infection is also aided by viral adaptation to host cellular pathways that reduce apoptosis and sustain long-term replication (Zhang et al., 2021). However, the pattern and duration of viral shedding from infected rodents remain highly variable depending on the hantavirus host system and stage of infection (Bagamian et al., 2012; Hardestam et al., 2008). A study on bank voles infected with Puumala hantavirus reported the detection of viral RNA in saliva, urine, and feces during defined periods of infection (Hardestam et al., 2008). Evidence obtained from naturally infected deer mice maintained in outdoor enclosures suggested that indirect environmental transmission may be less efficient than previously assumed (Bagamian et al., 2012).

Aerosolization of contaminated rodent excreta is the primary route of transmission of the causative virus to humans, particularly in enclosed settings (Vial et al., 2023). The intensity of virus shedding varies with rodent age, infection stage, season, and ecological factors, as these factors determine the efficiency of viral dissemination (Bagamian et al., 2012; Douglas et al., 2022; Hardestam et al., 2008). Seasonal breeding enhances the number of juvenile rodents that are susceptible to infection, which expedites the process of recurrent amplification of hantavirus transmission, particularly in peak hours (Goodfellow et al., 2021). The ecological dynamics of rodent populations are affected by rainfall, vegetation growth, and availability of food resources (Chen et al., 2021). Climatic conditions that increase food availability can eventually trigger surges in rodent populations, which further increase viral circulation in reservoir communities (Tian and Stenseth, 2019). In fact, extreme climate events such as El Niño can alter the breeding cycle of rodents and indirectly increase the risk of outbreaks by aiding in the rapid expansion of the rodent reservoir (Ashique et al., 2022).

Similarly, an increase in rodent density can facilitate the transmission of hantavirus through aggressive interactions, biting habits, and close contact with other hosts (Chen et al., 2021). Moreover, juvenile dispersal and territorial overlap play a vital role in sustaining the endemic transmission of the virus (Juan et al., 2019). It is important to note that, despite a reported decline in biodiversity, there is a rise in the dominance of efficient reservoir species, primarily due to reduced ecological competition and regulation by predators (Sánchez et al., 2021). In situations where simplified ecosystems are present, there is an increase in rodent–human interactions and a higher opportunity for zoonotic spillover (Plowright et al., 2021). Finally, habitat fragmentation and anthropogenic changes in the environment can selectively improve the survival of rodent species that are competent to sustain hantavirus infection circulation (Camp et al., 2021).

## 4 Climate change as a driver of hantavirus emergence

Climate change has been acknowledged as one of the key determinants for hantavirus outbreaks in different parts of the world (Ashique et al., 2022; Douglas et al., 2022; Ecke et al., 2022; Guterres and de Lemos, 2018; Karbalaei et al., 2022; Kell, 2022; Koehler et al., 2022; Moirano et al., 2024; Park et al., 2021; Prist et al., 2021; Tariq and Kim, 2022; Warner et al., 2021). Rising global temperatures due to the greenhouse effect have affected the reproductive cycles, survival rates, and population density of rodents, creating more opportunities for hantavirus amplification within reservoir hosts (Douglas et al., 2022). Similarly, altered precipitation patterns, especially increased rainfall following periods of drought, have resulted in an increase in vegetation growth and food availability, thereby indirectly promoting the expansion of rodent populations (Ecke et al., 2022). Environmental stressors, such as drought, can change rodent foraging behaviors and their proximity to human habitats, which may increase the potential for human–wildlife contact (Ashique et al., 2022). On the contrary, during floods, rodent populations are displaced from their natural habitats into peri-domestic settings, which then become the source of human–rodent interactions (Douglas et al., 2022).

Climate-driven habitat degradation and deforestation activities have also forced reservoir rodents to migrate into agricultural and urbanized areas, as highlighted in a systematic review (Moirano et al., 2024). Similarly, the expansion of peri-urban ecosystems has aided the formation of an ecological interface at which infected rodents can coexist with human populations (Douglas et al., 2022). Moreover, land-use changes by humans coupled with climate stress may alter rodent movement patterns and habitat overlap, potentially creating conditions that facilitate the spillover of infection from rodents to humans (Kell, 2022; Moirano et al., 2024). While environmental temperature and humidity are hypothesized to influence hantavirus persistence outside rodent hosts, the exact duration of infectivity in peri-domestic settings remains a subject of ongoing investigation (Guterres and de Lemos, 2018). In fact, cool and humid conditions tend to prolong the persistence of the virus in rodent excreta, thereby increasing the likelihood of aerosol-mediated transmission of infection (Koehler et al., 2022). Simultaneously, climate stress can modify the replication dynamics of the causative virus in rodents, which eventually alters shedding patterns and transmission intensity (Bagamian et al., 2012; Kell, 2022).

Warm climate conditions have played a vital role in the expansion of rodent reservoirs into previously unsuitable ecological regions and higher altitudes (Camp et al., 2021). This redistribution of rodents could potentially increase exposure risks among immunologically naïve human populations (Karbalaei et al., 2022; Kim et al., 2021). Andes virus outbreaks in South America have been linked to climatic conditions, where ecological alterations and habitat shifts may influence the overall transmission dynamics (Douglas et al., 2022; Prist et al., 2021). Molecular studies have suggested that ecological attributes might influence the evolution and endemicity of Andes virus in these areas (Warner et al., 2021). Similarly, for Sin Nombre virus (the most common agent of hantavirus cardiopulmonary syndrome in humans), climatic attributes, such as increased precipitation and vegetation growth, have been positively linked to periodic increases in deer mouse populations (Goodfellow et al., 2021). Climate change has also influenced rat population dynamics in Europe and East Asia and altered seasonal transmission cycles (Park et al., 2021; Tariq and Kim, 2022).

These observations are consistent with current evidence suggesting that climate change affects the transmission of hantavirus through multiple interconnected biological pathways rather than a single environmental factor (Ramos-Nino et al., 2026). In other words, temperature, precipitation, habitat fragmentation, and altered food availability cumulatively alter rodent population dynamics, viral shedding, and opportunities for human exposure (Kumar and John, 2026). However, the reported magnitude of these effects varies across geographical regions, likely due to differences in reservoir species, natural climate, and land-use patterns (Kumar and John, 2026; Ramos-Nino et al., 2026). This highlights that climate change acts primarily as an ecological amplifier of existing transmission cycles rather than as an isolated determinant of disease emergence (Kumar and John, 2026; Park et al., 2021; Ramos-Nino et al., 2026; Tariq and Kim, 2022).

## 5 Climate-induced ecological stress and viral evolution

Climate-related factors influence hantavirus dynamics in multiple ways, driving population-level viral amplification, while simultaneously shaping patterns of viral mutation, reassortment, and molecular evolution. High-density rodent populations augment viral amplification through close interactions, territorial overlap, and infected excreta in the environment (Goodfellow et al., 2021; Kim et al., 2021). Furthermore, high reservoir population densities may account for rapid viral transmission and increased replication cycles within local host populations, which expands genetic diversity through mutation and selection pressures (Ramos-Nino et al., 2026). Habitat destruction and alterations in land use can change the distribution, movement, and aggregation of reservoir hosts, thereby potentially increasing contact among previously separated host species and creating opportunities for cross-species virus transmission and co-infection (François et al., 2023; Kumar and John, 2026; Yin et al., 2025). This increased contact and co-infection may subsequently facilitate vital genetic exchange, including recombination and, in segmented viruses, reassortment (Gottdenker et al., 2014; Yin et al., 2025). The available evidence from wildlife supports this ecological-evolutionary link, with landscape changes linked to increased viral assortment in avian influenza and habitat sharing among wild bird species associated with cross-species transmission and the occurrence of recombinant viruses (François et al., 2023; Yin et al., 2025). These adaptive mutations and genetic changes may improve viral replication efficiency and environmental stability, as well as increase shedding intensity and persistence of the virus within rodent populations for longer periods of time (Goodfellow et al., 2021; Kell, 2022; LaTourrette and Garcia-Ruiz, 2022). These climate-linked selective pressures can aid in the effective replication of some specific strains across temperature ranges (Han and Worobey, 2011; LaTourrette and Garcia-Ruiz, 2022).

Habitat alterations may force rodent reservoirs into new ecological niches and peri-domestic areas, augmenting contact between distinct species and increasing the potential risk of cross-species spillover events (Camp et al., 2021; Kim et al., 2021). Further, climate-related environmental disruption can augment the incidence of spillover events through increased aerosolized exposure to infected rodent excreta in human habitats (Gorris et al., 2025; Jiang et al., 2017). Rapid environmental changes might expedite the process of molecular evolution of hantaviruses by increasing replication opportunities and facilitating host switching (Mull et al., 2022). The ongoing ecological disruption might accelerate the selection of specific hantavirus strains that are capable of more efficient transmission in rodents and humans (Zhang et al., 2021). Recent reviews have suggested that ecological disturbances resulting from climate change can increase opportunities for viral adaptation by promoting host switching, persistence in the environment for longer periods of time, and higher human contact with reservoirs (Fabbri et al., 2025; Munir et al., 2021; Vial et al., 2023). Moreover, although the association between environmental change and hantavirus emergence is on the rise, important uncertainties remain regarding the relative contribution of individual climatic variables to viral evolution in heterogeneous ecological settings.

## 6 Human encroachment, urbanization, and spillover interfaces

Deforestation tends to disrupt natural ecological barriers due to which rodents move into closer proximity with human settlements, increasing the risk of spillover of viruses (Mull et al., 2022). Similarly, large-scale land-use change has altered biodiversity patterns, which has favored rodent species to effectively maintain transmission cycles, as envisaged in a systematic review (Moirano et al., 2024). Large-scale land-use changes, infrastructure development, and urban expansion can alter rodent habitats and modify human-rodent interfaces, potentially influencing the spatial distribution and transmission risk of hantaviruses (Griffiths et al., 2022). Intensification of agriculture has resulted in an abundance of food resources that can facilitate the expansion of rodent populations in farming lands (Kazasidis and Jacob, 2023). Rodents in peri-urban locations have quickly adapted and are often abundant in grain storage facilities and livestock areas, enabling the persistent circulation of the virus near human dwellings (Liu et al., 2023). Agricultural activities and changes in food availability can influence rodent abundance and distribution, potentially modifying human-rodent contact and the risk of hantavirus transmission (Liu et al., 1984; Moirano et al., 2024).

Personnel employed in agriculture, forestry, the military, and construction sites are at high risk of exposure to hantaviruses owing to their frequent contact with rodent-infested environments (Krug et al., 2023; Riccò et al., 2021). Recreational activities in rural and wilderness areas, including camping and overnight stays in rodent-infested cabins or shelters, have also been associated with hantavirus exposure in endemic regions (de St Maurice et al., 1993; Núñez et al., 2014). Extreme climate events may simultaneously displace rodent populations and humans into shared shelters, potentially increasing opportunities for zoonotic transmission (Ferro et al., 2020). Rapid urbanization and overcrowding can facilitate rodent infestation and environmental contamination in densely populated communities, (Liu et al., 1984; Moirano et al., 2024) and the presence of poor sanitation, inadequate waste management, and informal settlements can provide favorable ecological conditions for rodent survival and maintenance of the virus (Ashique et al., 2022; Griffiths et al., 2022).

An important concern of the current evidence is that much of the available literature includes ecological analyses, observational studies, surveillance reports, and modeling investigations (Romeo et al., 2025). These study designs can demonstrate the significance of identifying associations but have no role in ascertaining direct causal relationships between climate variables and the emergence of hantavirus (Fabbri et al., 2025). Moreover, owing to the differences in surveillance intensity, diagnostic capacity, geographical coverage, and reporting systems, significant heterogeneity has been reported among published studies (Ramos-Nino et al., 2026). There is a need to invest in carrying out multidisciplinary investigations, aiming to integrate long-term ecological monitoring, genomic surveillance, climate modeling, and experimental studies, which will be vital for improving understanding and strengthening evidence-driven preparedness strategies (Guzzi et al., 2026). Additionally, integrating ecological surveillance with genomic epidemiology can improve climate-informed early warning systems, further strengthening outbreak prediction and facilitating the timely implementation of public health interventions (Guzzi et al., 2026).

## 7 Conclusion

To conclude, climate change has significantly influenced the ecology, transmission dynamics, and molecular evolution of hantaviruses by changing the behavior of rodents, their habitats, and the environmental persistence of viral particles. These interconnected ecological and virological processes have created opportunities for viral amplification in reservoir hosts and increased the risk of zoonotic spillover into human populations. Translating this in-depth information into practice will require the integration of climate-informed ecological surveillance, genomic epidemiology, and predictive modeling with holistic One Health frameworks. These interdisciplinary approaches can strengthen early warning systems, provide evidence for the formulation of public health policies, improve preparedness for climate-sensitive zoonotic diseases, and aid targeted interventions in high-risk areas. This article contributes to Sustainable Development Goal 3 by highlighting climate-sensitive zoonotic disease risks and advocating integrated surveillance, early warning systems, and preparedness strategies for preventing hantavirus outbreaks. It also supports Sustainable Development Goal 13 (Climate Action) and Sustainable Development Goal 15 (Life on Land) by emphasizing the health consequences of climate change, habitat disruption, biodiversity loss, and altered wildlife–human interfaces.

## Data Availability

The original contributions presented in the study are included in the article/supplementary material, further inquiries can be directed to the corresponding author.
